# Prevalent and Incident Bacterial Vaginosis Are Associated with Sexual and Contraceptive Behaviours in Young Australian Women

**DOI:** 10.1371/journal.pone.0057688

**Published:** 2013-03-05

**Authors:** Catriona S. Bradshaw, Jennifer Walker, Christopher K. Fairley, Marcus Y. Chen, Sepehr N. Tabrizi, Basil Donovan, John M. Kaldor, Kathryn McNamee, Eve Urban, Sandra Walker, Marian Currie, Hudson Birden, Francis Bowden, Suzanne Garland, Marie Pirotta, Lyle Gurrin, Jane S. Hocking

**Affiliations:** 1 Melbourne Sexual Health Centre, Melbourne, Victoria, Australia; 2 Sexual Health Unit, School of Population Health, University of Melbourne, Melbourne, Victoria, Australia; 3 Department of Epidemiology and Preventive Medicine, Monash University, Melbourne, Victoria, Australia; 4 Centre for Excellence in Rural Sexual Health, Melbourne Medical School, Rural Health Academic Centre, University of Melbourne, Melbourne, Victoria, Australia; 5 Centre for Women’s Health, Gender and Society, Melbourne School of Population Health, University of Melbourne, Melbourne, Victoria, Australia; 6 Molecular Microbiology Department, the Department of Microbiology and Infectious Diseases, the Royal Women’s Hospital, Melbourne, Victoria, Australia; 7 Department of Obstetrics and Gynaecology, University of Melbourne, Melbourne, Victoria, Australia; 8 Kirby Institute, University of New South Wales, and Sydney Sexual Health Centre, Sydney Hospital, Sydney, New South Wales, Australia; 9 Family Planning Victoria, Melbourne, Victoria, Australia; 10 Monash Medical Centre, Department of Obstetrics and Gynaecology, Melbourne, Victoria, Australia; 11 Australian National University, Canberra, Australian Capital Territory, Australia; 12 University Centre for Rural Health, and Sydney Institute for Emerging Infectious Diseases & Biosecurity, Sydney School of Public Health, The University of Sydney, Lismore, New South Wales, Australia; 13 Primary Care Research Unit, Department of General Practice, University of Melbourne, Melbourne, Victoria, Australia; 14 Centre for Molecular, Environmental, Genetic and Analytic Epidemiology, School of Population Health, University of Melbourne, Melbourne, Victoria, Australia; Institute for Genome Sciences, University of Maryland School of Medicine, United States of America

## Abstract

**Background:**

To determine prevalence and incidence of bacterial vaginosis (BV) and risk factors in young sexually-active Australian women.

**Methods:**

1093 women aged 16–25 years were recruited from primary-care clinics. Participants completed 3-monthly questionnaires and self-collected vaginal smears 6-monthly for 12-months. The primary endpoint was a Nugent Score = 7–10 (BV) and the secondary endpoint was a NS = 4–10 (abnormal flora [AF]). BV and AF prevalence estimates and 95% confidence intervals (95%CI) were derived, and adjusted odds ratios (AOR) calculated to explore epidemiological associations with prevalent BV and AF. Proportional-hazards regression models were used to examine factors associated with incident BV and AF.

**Results:**

At baseline 129 women had BV [11.8% (95%CI: 9.4–14.2)] and 188 AF (17.2%; 15.1–19.5). Prevalent BV was associated with having a recent female partner [AOR = 2.1; 1.0–4.4] and lack of tertiary-education [AOR = 1.9; 1.2–3.0]; use of an oestrogen-containing contraceptive (OCC) was associated with reduced risk [AOR = 0.6; 0.4–0.9]. Prevalent AF was associated with the same factors, and additionally with >5 male partners (MSP) in 12-months [AOR = 1.8; 1.2–2.5)], and detection of *C.trachomatis* or *M.genitalium* [AOR = 2.1; 1.0–4.5]. There were 82 cases of incident BV (9.4%;7.7–11.7/100 person-years) and 129 with incident AF (14.8%; 12.5–17.6/100 person-years). Incident BV and AF were associated with a new MSP [adjusted rate ratio (ARR) = 1.5; 1.1–2.2 and ARR = 1.5; 1.1–2.0], respectively. OCC-use was associated with reduced risk of incident AF [ARR = 0.7; 0.5–1.0].

**Conclusion:**

This paper presents BV and AF prevalence and incidence estimates from a large prospective cohort of young Australian women predominantly recruited from primary-care clinics. These data support the concept that sexual activity is strongly associated with the development of BV and AF and that use of an OCC is associated with reduced risk.

## Introduction

Bacterial vaginosis (BV) is the most common cause of abnormal vaginal discharge in women of reproductive age. However, internationally, most BV prevalence and incidence data are derived from testing women attending sexual/reproductive services, rather than from community-based screening studies. The widely cited 2001-4 U.S. National Health and Nutrition Survey (NHANES) showed 29% of 14–49 year old women had BV, with higher prevalence among African-American (51.6%) compared to Hispanic (32.1%) and Caucasian (23.2%) women [1]. While a study of asymptomatic women attending general practices in the United Kingdom, and a study of Australian university students, reported notably lower prevalence estimates of 9% and 5% respectively [2], [3]. Some of the highest community-based estimates have been reported from rural sub-Saharan Africa, with a systematic review of women attending antenatal facilities finding pooled BV prevalence estimates of 51% in East/Southern Africa and 38% in West/Central Africa [4].

BV incidence studies have generally been conducted in high prevalence populations, with a high proportion of participants from STI clinics or from disadvantaged backgrounds. These studies are likely to provide higher BV incidence estimates compared with community-based samples and have tended to report rates in excess of 20/100 woman-years [5], [6], [7], [8], [9], [10], [11], [12]. In contrast, a prospective study of 17–21 year old Australian university students reported a low incidence rate of 2.2/100 woman-years in sexually-active participants, and no incident BV in sexually-inactive women [13].

While the aetiology of BV and whether it is sexually transmitted remains unclear, epidemiological studies show a consistent association between BV and sexual activity. Some of the strongest evidence comes from a meta-analysis of 43 studies which found prevalent and incident BV were associated with new or multiple male partnerships, report of female partners and inconsistent condom use [14]. Observational studies have, however, also reported associations between increased risk of BV and non-sexual behavioural practices such as smoking [15] and vaginal douching/cleaning practices [16], and a number of cross-sectional and longitudinal studies have shown a reduced risk of BV in women using hormonal contraceptives [6], [17], [18], [19], [20], [21]. Current antibiotic regimens for BV have limited effectiveness with up to 60% of women experiencing recurrence within 12 months [17]. Improving our understanding of how behavioural factors are associated with the development and recurrence of BV is integral to developing more effective approaches for the prevention and treatment of BV.

We present BV prevalence and incidence estimates, derived using the Nugent method, and examine the associated risk factors for the development of BV and abnormal flora (AF), in a large cohort study of young sexually-active women predominantly recruited from primary-care clinics. These are the first community-based estimates for the Australian population.

## Methods

### Recruitment, Clinical and Laboratory Methods

Women aged 16 to 25 years attending 29 primary-care services (general practice/family planning/sexual health services) in three Australian states, for any reason, were invited to participate in a cohort study to determine *C.trachomatis* incidence and re-infection rates (detailed methods described elsewhere) [22]. Participants were also invited to enroll in a sub-study to determine the prevalence and incidence of BV and *Mycoplasma genitalium*. Women were eligible if they were sexually-active, aged 16–25 years, not pregnant and contactable by post. Research assistants approached consecutive eligible women at clinic sites and obtained written informed-consent. Participants completed 3-monthly questionnaires on demographic, sexual and behavioural data, recent antibiotic and contraceptive use, and genital symptoms. They self-collected a vaginal swab and smeared the sample onto a glass slide in clinic at baseline, and then at home at 6 and 12 months. Samples and questionnaires were returned by post.

BV was defined as a Nugent score (NS) of 7–10, intermediate flora as a NS = 4–6, and normal flora as a NS = 0–3 [23]. Abnormal flora (AF) was defined as a NS = 4–10. All slides were scored by an experienced microbiologist. An independent senior microbiologist reread all NS 6 and 7 slides, had 50 problematic slides referred to her for a second opinion, and additionally randomly audited 10% of slides in each Nugent category. Concordance in the random audit was 92%; 9/112 slides were found to have a minor discrepancy defined as a difference of ≥2 in Nugent score that did not result in misclassification of BV, or normal versus intermediate flora. Use of self-collected samples for BV diagnosis has been shown to be comparable to clinician-collected samples [24]. Women with symptomatic BV were treated with metronidazole 400 mg orally twice daily or 2%-clindamycin cream vaginally for 7 nights.

Participants were tested for *C.trachomatis* and *M.genitalium* at baseline and during follow-up by PCR using the Cobas-TaqMan CT-assay (Roche Applied Science) and a PCR-assay targeting the 517 bp region of the 16S rRNA gene of *M.genitalium*, respectively [25]. Detailed laboratory methodology and results have been described elsewhere [22], [26].

### Statistical Methods

Data were analysed using STATA 12.0 (StataCorp LP,College Station,USA). Assuming a design effect of 2 to take account of within-clinic correlation, a sample size of 1000 women was sufficient to generate 95% confidence intervals (CIs) of 7.2–12.8/100 person-years if the estimated incidence rate was 10/100 person-years.

BV and AF prevalence estimates and 95% CIs were calculated adjusting for within-clinic correlation. Adjusted odds ratios (AOR) and robust standard errors were calculated to explore epidemiological associations of variables with the primary outcome, prevalent BV (NS 7–10 versus NS 0–6), and secondary outcome, AF (NS 0–3 versus NS 4–10).

Women with a NS<7 at baseline were included in the cohort analysis. Incident BV was defined as the first occurrence of a NS = 7–10 in a participant with a previous NS<7; this led to a participant being censored from the incidence analysis. The association between risk factors and incident BV was investigated using a discrete-time version of the proportional-hazards regression model [27]. Variables were included in the model on the basis of the likelihood ratio test, or if they had been associated with BV in previous studies. Follow-up concluded 18 months after enrolment. The same statistical methods were applied to examination associations with incident AF.

Written informed consent was obtained from all participants. All eligible women were assessed for competency by research staff prior to being invited into the study. The research staff worked closely with the clinical staff to ensure only competent women were approached. All ethics committees approved the inclusion of participants over the age of 16 without parental or guardian consent. Ethics approval to conduct this study was obtained from ten Human Research Ethics Committees throughout Australia including: The Australian Capital Territory (ACT) Health Human Research Ethics Committee, ACT; The Alfred Health Ethics Committee, Melbourne; The University of Ballarat Ethics Committee, Ballarat, Victoria; Family Planning Victoria Human Research Ethics Committee, Victoria; The North Coast Area Health Service, Human Research Ethics Committee, New South Wales (NSW); The South Eastern Sydney Area Health Service, Human Research Ethics Committee - Northern Area, NSW; The University of Melbourne, Human Research Ethics Committee, Victoria; The University of Newcastle Human Research Ethics Committee, NSW; The University of NSW Human Research Ethics Committee, NSW; Family Planning NSW Research Ethics Committee, NSW [22], [26].

## Results

### Demographic and Behavioural Characteristics of the Study Population

Overall, 1116 women aged 16 to 25 years were recruited from 29 clinic-sites across Australia, with an overall response rate of 66% [22], [26]. A total of 1110 women (99.5%) agreed to participate in the BV sub-study. Of these 1093 (98.5%) provided a readable baseline slide and behavioural data, and were included in prevalence analyses. Nine hundred and sixty-four (88.2%) women with a baseline NS<7 continued in the cohort study, provided ≥1 slides and behavioural data, and contributed 871.20 person years of follow-up to incidence analyses.

Participants had a median age of 21 years, and were predominantly recruited from general practice (66%), Table 1. The majority (88%) were Australian-born, 24 (2%) were Aboriginal or Torres Strait Islander, 471 (44%) had completed tertiary studies, and 408 (38%) were employed. At baseline 638 (58%) women were using hormone-based contraception: 567 (89%) used the combined oral contraceptive pill (COCP), 5 (1%) an intra-vaginal ring, and 66 (10%) a progesterone implant or depot-injection. Seven women had an intra-uterine device; whether it was a levo-norgesterel device was unknown. In the prior year, one third of participants had ≥3 male sexual partners (MSP) and 103 (9%) ≥1 female partners (FSP).

**Table 1:** Demographic and behavioural associations with prevalent bacterial vaginosis by univariate and multivariate analysis (n = 1093).

Variable	N (%)	Prevalence (95%CI)	Unadjusted OR^a^ (95% CI)	Adjusted OR^a,b^ (95% CI)
**Age**				
** 16–20**	455 (41.6)	11.4 (9.0, 13.9)	1.0	
** 21–25**	638 (58.4)	12.3 (9.3, 15.3)	1.1 (0.8, 1.5)	
**Recruitment site**				
** General practice**	726 (66.4)	9.9 (7.7, 12.1)	1.0	1.0
** Sexual health/family planning**	367 (33.6)	15.5 (11.8, 19.3)	1.7 (1.1, 2.5)	1.2 (0.8, 1.7)
**Country of birth**				
** Australian born**	916 (88.4)	10.5 (8.5, 12.5)	1.0	
** Not Australian born**	120 (11.6)	15.8 (9.2, 22.4)	1.6 (1.0, 2.7)	
**Aboriginal/Torres Strait Islander^c^**				
** No**	1038 (97.7)	6.9 (0.0, 26.8)	1.0	
** Yes**	24 (2.3)	11.3 (9.3, 13.2)	1.1 (0.3, 3.7)	
**Highest education status**				
** Completed tertiary studies**	593 (55.7)	7.2 (4.9, 9.6)	1.0	1.0
** Completed secondary studies**	471 (44.3)	14.5 (11.7, 17.3)	2.2 (1.4, 3.5)	1.9 (1.2, 3.0)
**Employment status**				
** Unemployed**	408 (38.3)	12.0 (8.8, 15.2)	1.0	
** Employed**	656 (61.7)	11.0 (8.6, 13.4)	0.9 (0.7, 1.3)	
**Number of male sex partners in prior 12 months**				
** 0–2**	704 (67.2)	9.4 (6.9, 11.9)	1.0	1.0
** 3–4**	207 (16.4)	12.6 (8.0, 17.1)	1.4 (0.8, 2.3)	1.3 (0.7, 2.2)
** 5+**	136 (7.2)	18.4 (10.0, 26.7)	2.2 (1.2, 3.9), p<0.01^d^	1.6 (0.8, 3.1), p = 0.12^d^
**Any female sex partners in prior 12 months**				
** No**	990 (90.6)	10.5 (8.4, 12.6)	1.0	1.0
** Yes**	103 (9.4)	24.3 (13.8, 34.7)	2.7 (1.6, 4.7)	2.1 (1.0, 4.4)
**Current use of oestrogen-containing contraception**				
** No**	497 (46.5)	14.5 (11.4, 17.6)	1.0	1.0
** Yes**	572 (53.5)	8.7 (6.3, 11.2)	0.6 (0.4, 0.8)	0.6 (0.4, 0.9)
**Coinfection with ** ***C.trachomatis or M.genitalium***				
** No**	1014 (92.8)	1.1 (8.6, 13.7)	1.0	1.0
** Yes**	79 (7.2)	20.2 (8.7, 31.8)	2.0 (0.9, 4.4)	2.0 (0.9, 4.6)
**Self-reported history of BV**				
** No**	1007 (94.7)	11.1 (8.8, 13.5)	1.0	
** Yes**	56 (5.3)	16.1 (6.7, 25.4)	1.5 (0.8, 3.1)	
**Antibiotic use in last 2 months**				
** No**	785 (73.5)	12.1 (9.3, 14.8)	1.0	
** Yes**	285 (26.5)	9.2 (0.1, 12.5)	0.7 (0.5, 1.1)	
Abnormal vaginal discharge*				
** No**	822 (77.0)	10.1 (7.8, 12.2)	1.0	
** Yes**	246 (23.0)	15.4 (10.7, 20.2)	1.6 (1.1, 2.4)	
Abnormal vaginal odour*				
** No**	884 (82.8)	8.9 (7.0, 10.9)	1.0	
** Yes**	184 (17.2)	22.8 (16.9, 28.8)	3.0 (2.2, 4.1)	
**Baseline Nugent score**				
** 0–3**	905 (82.8)	82.8 (80.1, 85.5)		
** 4–6**	59 (5.4)	5.4 (4.0, 6.8)		
** 7–10**	129 (11.8)	11.8 (9.4, 14.2)		

a = odds ratio; b = multivariate analysis including: recent female sex partner, current use of oestrogen containing contraception, numbers of male sex partners, recruitment site (sexual health/family planning clinic or general practice clinic), education level achieved, tested positive for chlamydia at baseline; c = Identify as Aboriginal and/or Torres Strait Islander Origin; d = test for trend; * self-reported and note clinical symptoms are not included in any multivariate analyses.

### Baseline Clinical and Laboratory Characteristics of the Study Population

**Figure 1:**
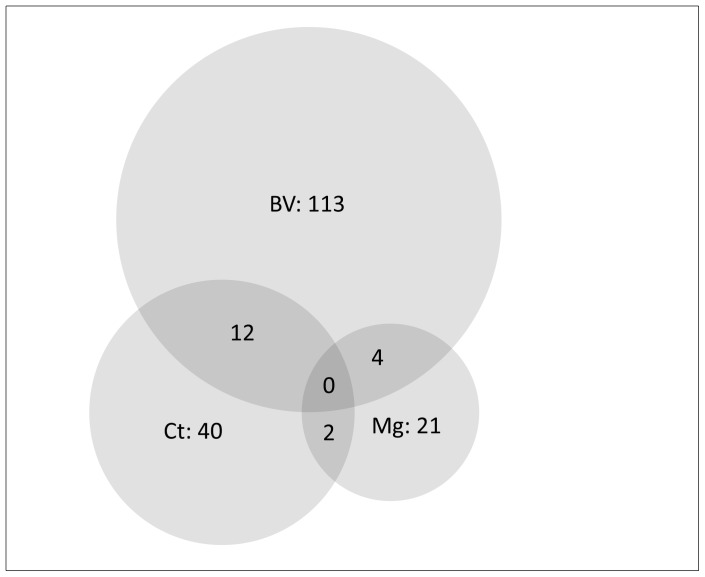
Cases of bacterial vaginosis, *Chlamydia trachomatis* and *Mycoplasma genitalium* at recruitment and co-infections.

At baseline, 129 women had a NS of 7–10 [11.8% (95%CI: 9.4–14.2)], Table 1. Fifty-nine women had intermediate flora (5.4%; 4.0–6.8), and 905 had normal flora (82.8%; 80.1–85.5). The prevalence of AF was 17.2% (15.1–19.5). At recruitment, 246 (23%) women reported an abnormal vaginal discharge and 184 (17%) an abnormal odour. Twelve women had concurrent BV and *C.trachomatis*[1.1%; 0.5–1.7)], and 4 concurrent BV and *M.genitalium*[0.4%; 0.0–0.7)], Figure 1.

### Factors Associated with Prevalent BV and AF

By univariate analysis, women with prevalent BV were more likely to be recruited from sexual health/family planning services (OR = 1.7; 1.1–2.5), to have no tertiary education (OR = 2.2; 1.4–3.5), to be born overseas (OR = 1.6; 1.0–2.7), and in the prior year to report a FSP (OR = 2.7; 1.6–4.7) and ≥5 new MSPs (OR = 2.2; 1.2–3.9), Table 1. Current use of an oestrogen-containing contraception (OCC, defined as COCP or ring) was associated with a lower prevalence of BV (OR = 0.6; 0.4–0.8), while use of any hormone-based method was not (OR = 0.8; 0.5–1.2). Co-detection of *C.trachomatis or M.genitalium* had a borderline association with BV (OR = 2.0; 0.9–4.4). Women with prevalent BV were more likely to report an abnormal vaginal discharge (OR = 1.6; 1.1–2.4) or odour (OR = 3.0; 2.2–4.1).

By multivariate logistic regression analysis, women with prevalent BV were more likely to have had a FSP in the prior year (AOR = 2.1; 1.0–4.4), and no tertiary education (AOR = 1.9; 1.2–3.0). OCC-use was associated with decreased odds of BV (AOR = 0.6; 0.4–0.9), and there was a borderline association with co-detection of *C.trachomatis or M.genitalium* [AOR = 2.0; 0.9–4.6), Table 1. Clinical symptoms were not included in multivariate analyses.

The findings for prevalent AF did not greatly differ from BV. Women with prevalent AF were more likely in the last year to have had a FSP [AOR = 1.9; 1.0–3.6)], and ≥5 new MSPs (AOR = 1.8; 1.2–2.5), to be co-infected with *C.trachomatis or M.genitalium* (AOR = 2.1; 1.0–4.5) and to have no tertiary education (AOR = 1.6; 1.2–2.3); OCC-use decreased the odds of AF(AOR = 0.7; 0.5–1.0).

### Incidence and Risk Factors Associated with BV and AF

There were 82 cases of incident BV [9.4 (95% CI: 7.7–11.7)/100 person-years], and incidence was stable during follow-up at 9.3 (95% CI: 5.8–14.9)/100 person years over the first 6 months and 9.4 (95% CI:7.4–12.0)/100 person years over the last 6 months.

By univariate analysis, incident BV was associated with being employed (RR = 1.4; 1.0–2.0), and having a new MSP during follow-up (RR = 1.6; 1.2–2.3), Table 2. OCC-use in the 3-months prior to testing was associated with lower rates of incident BV (RR = 0.7; 0.5–1.0); use of any hormonal contraceptive was not (RR = 0.8; 0.5–1.2). Women with incident BV were more likely to report genital symptoms in the interval prior to testing including an abnormal vaginal discharge (RR = 1.9; 1.3–2.7), odour (RR = 2.9; 2.0–4.1), or abdominal pain (RR = 1.5; 1.1–2.1). By multivariate analysis, having a new MSP during follow-up was associated with incident BV (ARR = 1.5; 1.1–2.2); there was a borderline reduction in the risk of incident BV with OCC-use (ARR = 0.7; 0.5–1.1), Table 2.

**Table 2:** Demographic and behavioural associations with incident bacterial vaginosis by univariate and multivariate analysis.

Characteristic	Rate per 100,000 womanyears (95%CI)	Unadjusted RR^a^ (95% CI)	Adjusted RR^b^ (95% CI)
**Age**			
** 16 to 20**	9.8 (7.4, 12.8)	1.0	
** 21 to 25**	8.9 (6.3, 12.6)	0.8 (0.6, 1.2)	
**Recruitment site**			
** General practice**	8.8 (6.7, 11.4)	1.0	1.0
** Sexual health/family planning**	10.9 (7.6, 15.6)	1.2 (0.9, 1.8)	1.2 (0.8, 1.8)
**Country of birth**			
** Australian born**	8.6 (6.8, 11.1)	1.0	
** Not Australian born**	11.3 (6.1, 21.0)	1.5 (0.9, 2.6)	
**Aboriginal/Torres Strait Islander^c^**			
** No**	9.1 (7.2, 11.3)	1.0	
** Yes**	9.2 (2.3, 36.6)	0.5 (0.1, 2.0)	
**Education status**			
** Completed secondary studies**	10.1 (7.4, 13.7)	1.0	
** Completed tertiary studies**	8.1 (5.9, 11.2)	1.1 (0.8, 1.6)	
**Employment status**			
** Not currently employed**	8.4 (5.8, 12.2)	1.0	1.0
** Employed**	9.5 (7.2, 12.5)	1.4 (1.0, 2.0)	1.4 (0.9, 2.0)
**Recent^d^ new male sexual partner**			
** No**	7.9 (6.0, 10.4)	1.0	1.0
** Yes**	14.1 (9.9, 20.2)	1.6 (1.2, 2.3)	1.5 (1.1, 2.2)
**Recent^d^ female sex partner**			
** No**	9.1 (7.3, 11.5)	1.0	
** Yes**	13.1 (6.5, 26.1)	1.5 (0.8, 2.5)	
**Use of oestrogen containing contraception**			
** No**	11.3 (8.4, 15.2)	1.0	1.0
** Yes**	8.0 (5.8, 10.9)	0.7 (0.5, 1.0)	0.7 (0.5, 1.1)
**Recent^d^** ***Mycoplasma genitalium*** ** infection**			
** No**	10.1 (8.1, 12.4)	1.0	
** Yes**	6.9 (0.2, 33.9)	0.5 (0.1, 3.4)	
**Recent^d^** ***Chlamydia trachomatis*** ** infection**			
** No**	9.2 (7.4, 11.5)	1.0	
** Yes**	17.0 (6.4, 45.3)	1.5 (0.7, 3.4)	
**Antibiotic use in the last 2 months**			
** No**	8.3 (6.4, 10.8)	1.0	
** Yes**	13.5 (9.2, 19.8)	1.0 (0.7, 1.5)	

a = rate ratio; b = multivariate analysis including: recent new male sex partner, current use of oestrogen containing contraception, currently employed, recruitment site (sexual health/family planning clinic or general practice clinic); c = Aboriginal and/or Torres Strait Islander origin. d = recent refers to within the 3 months prior to testing.

There were 129 cases of incident AF during the study [14.8 (95% CI: 12.5–17.6)/100 person-years]. By multivariate analysis, having a new MSP during the study was associated with incident AF (ARR = 1.5; 1.1–2.0) and OCC-use was associated with a reduced risk of incident AF (ARR = 0.7; 0.5–1.0).

## Discussion

This study presents findings on the prevalence and incidence of BV and AF among a large community-based cohort of Australian women. In this young cohort we found that exposure to a new male partner was associated with increased risk of incident BV and AF, while use of an OCC was associated with a reduced risk. The young age and low BV prevalence in participants, together with the stable BV incidence throughout the study, all facilitated the study of the association between recent sexual and contraceptive behaviours and the development of incident BV. These are the first data to give an estimation of the prevalence (12%) and incidence (9.4/100 person years) of BV in young Australian women in the community. Our findings indicate that sexual exposure to new partners is influential in the development of BV and AF, and that OCC appears to be associated with a reduced risk.

The prevalence and incidence of BV in this large cohort of young Australian women is significantly lower than that reported from the NHANES data [1], and the majority of prospective studies in heterosexual women [5], [6], [7], [8], [9], [10], [11], [12]. This is likely to be due to the young age of our participants and the fact that the majority were recruited from primary-care compared with those in published studies. Our prevalence data are more in keeping with a UK study of women attending general practices for cervical cytology (9%) [2], but notably higher than that found in 17–21 year old Australian university students (5%) [3]. Our data supports the previously reported association between BV and lower levels of education, which is likely to be more broadly reflective of socio-economic and health inequity, and greater levels of sexual risk behaviour [1], [28].

Incident BV and AF, in this young predominantly heterosexual cohort, were associated with report of a new male partner. A number of investigators have reported an association between the development of BV and exposure to a new male partner [5], [7], [9]. In a study of women recruited from an STI service, sex with a new partner was the only behaviour associated with incident BV [7]. In a cohort of female sex workers in West Africa, Nagot reported having ≥3 male sex partners in the prior week was associated with incident BV [9]. Using cohort and case cross-over analytical methods to reduce unmeasured confounding, Gallo found the only consistent association with incident BV in high-risk women was detection of spermatozoa on Gram stain [29]. In our cohort increased numbers of male partners in the year prior to enrolment was also associated with increased odds of prevalent AF but not BV. It is interesting to note the association reported in the meta-analysis between new or multiple male partnerships and prevalent and incident BV was of similar magnitude to that found in the current cohort (RR = 1.6; 95% CI: 1-5-1.8) [14].

It was not only exposure to male partners that conferred an increased risk of BV in this community cohort, but report of female partners in the prior year doubled the odds of prevalent BV and AF. The association between increased BV risk and female partnerships is well known but poorly understood. A higher prevalence of BV is consistently reported in lesbians compared to heterosexual women within the same communities, and high levels of concordance of vaginal flora has been reported between monogamous female partners [30], [31], [32]. Again the magnitude of the effect observed in our cohort was strikingly similar to that found by meta-analysis which showed report of any female partner(s) doubled the odds of BV (RR = 2.0; 95% CI 1.7–2.3) [14]. While these data are compelling, the fundamental question as to whether sexual activity with male or female partners contributes to the development of BV through transmission of BV associated-bacteria, or instead is impacting adversely on vaginal colonization with protective *Lactobacillus* species, remains unanswered. The association between BV and increased detection of STIs, which has been reported in a number of studies [5], [33], [34] provides further support for the strong relationship between BV and sexual activity. However, whether BV increases susceptibility to STIs, or BV and STIs are co-infections that result from shared risk factors is unknown.

Use of an OCC in this cohort was associated with a reduced risk of prevalent BV and AF, and incident AF, after adjusting for confounding factors including numbers of sexual partners. This predominantly reflected COCP use in this cohort, and when use of any hormonal contraceptive, which included progesterone-only methods, was examined there was no significant association. This association has been evident in a number of published studies. Hormonal contraceptives, mainly combined methods, have been reported to be protective against prevalent [18], [19], [20], incident [6], [35] and recurrent [17], [19], [21] BV. While there are a number of confounding factors that may explain this association it is consistently evident in the literature, and many analyses including our own, have adjusted for known confounders such as numbers of sexual partners and educational level. There are also biologically plausible explanations for this apparent association. It has been hypothesized that oestrogen increases the glycogen-content of epithelial cells, a substrate for *Lactobacillus* species to generate lactic acid, which appears to be a potent inhibitor of BV-associated bacteria [36]. It is possible that contraceptive use influences the vaginal immune response, with Cherpes reporting an association between hormonal contraceptive use and altered vaginal immunity in BV [37]. Clearly further studies are need to determine if and how OCC may provide protection against BV or whether the observed association is due to unmeasured confounding.

There were a number of limitations to our study. Participants were more likely to be Australian-born, better educated, and more sexually-active than the background Australian population of this age [22], [38]. As a consequence, our study may not be representative of all Australian 16–25 year old women. Two thirds of recruits were derived from general practice and the remainder from sexual health/family planning services. BV prevalence was slightly higher in recruits from family planning/sexual health services compared to general practices, however, the BV incidence did not differ between these sites, and care was taken to adjust all analyses for clinic type. The inclusion of sexual health/family planning services in this cohort may have some impact on the generalizability of prevalence and incidence estimates, however, it is less likely to effect the measures of association (relative risks). Overall, 80–90% of young Australian women visit a general practice clinic each year [39], so this study population should provide a reasonable sample of young women in the community in this age group. Another important limitation is that the precision of our estimates of BV incidence reflect the availability of event data, which was constrained by six-monthly sample collection. Studies employing Gram stain analysis and more sensitive molecular methods, such as quantitative PCR and pyrosequencing, have shown that the vaginal microbiota can be highly dynamic. Dramatic changes in lactobacillus and BV-associated bacterial species, and rapid fluctuation in Nugent scores can occur in some individuals over a menstrual cycle, although longitudinal studies have also shown that vaginal bacterial communities can be more stable in some women than others [40], [41], [42], [43], [44], [45]. These studies indicate that factors such as menstruation and sexual behaviours may influence the stability of the vaginal microbiota in women, however the clinical significance of these fluctuations is not currently known. Importantly, the broad sampling interval in this study means cases of BV could have been missed, which may have lead to an under-estimate of the incidence rate. Of note, there was a significant association between self-report of abnormal vaginal discharge and/or odour and diagnosis of incident BV by the Nugent method, indicating that cases of incident infection that were detected were more likely to represent clinically significant BV.

Overall the strengths of this study include the diverse range of geographical location and socio-economic status from which participants were recruited, the high recruitment and retention rates, and inclusion of a high proportion of under 21 year old women (42%), a group that is difficult to engage in research. A further strength is that the young age, the low BV prevalence, and the stable incidence of BV throughout the study all facilitated the study of the relationship between recent behaviours and incident BV.

### Conclusion

These are the first data to give an estimation of the prevalence and incidence of BV in young Australian women in the community. The consistent manner in which sexual risk factors are identified in association with BV, in populations that differ widely in their geographical and socioeconomic circumstances, lends considerable weight to the concept that sexual activity with both men and women is highly influential if not integral to the development of BV. However, it appears likely that sexual and contraceptive practices influence not just the development of BV and AF but also recurrence. A recent treatment trial showed no benefit from combining oral metronidazole with vaginal clindamycin over oral metronidazole alone. BV recurrence in this cohort was instead strongly associated with remaining with the same pre-treatment sexual partner and inconsistent condom use, while use of oestrogen-containing contraceptives was associated with a reduced risk of recurrence [17], [19], [21]. Debate will continue as to whether the consistent association between BV and sexual activity is a direct result of transmission of putative agent(s) that have an aetiological role in the development and recurrence of BV, or whether sexual activity itself impacts adversely on vaginal colonization with protective *Lactobacillus* species thereby facilitating BV development and recurrence. We are only just beginning to understand the diversity of the vaginal microbiota in healthy women [41], and have yet to fully understand the effects of sexual behaviours on it, and if and how use of hormone-based contraceptives may influence a woman’s vaginal microbiota and susceptibility to BV. Clearly, identifying potentially modifiable behaviours that are associated with the development and recurrence of BV within large cohort studies is an integral step to the development and evaluation of targeted interventions to improve prevention and treatment approaches for BV.
